# Limited Prognostic Value of Psoas Muscle Indices in Patients Undergoing Revascularization for Chronic Limb-Threatening Ischemia

**DOI:** 10.3390/medsci13040227

**Published:** 2025-10-12

**Authors:** Joanna Halman, Jakub Dybcio, Kamil Myszczyński, Nina Kimilu, Agnieszka Blacha, Grzegorz Owedyk, Jacek Wojciechowski, Mariusz Siemiński

**Affiliations:** 1 Vascular Surgery Department, Medical University of Gdańsk, University Clinical Centre in Gdańsk, 80-952 Gdańsk, Poland; jacek.wojciechowski@gumed.edu.pl; 2 Student Scientific Circle of Vascular Surgery, Faculty of Medicine, Medical University of Gdańsk, 80-210 Gdańsk, Poland; dybcioj@gmail.com; 3 Centre of Biostatistics and Bioinformatics Analysis, Medical University of Gdansk, 80-210 Gdansk, Poland; kamil.myszczynski@gumed.edu.pl; 4 Department of Clinical Nutrition and Dietetics, Medical University of Gdansk, 80-210 Gdansk, Poland; nina.kimilu@gumed.edu.pl; 5 Scientific Circle of Neurotraumatology, Department of Emergency Medicine, Medical University of Gdańsk, 80-210 Gdańsk, Poland; a.blacha@gumed.edu.pl (A.B.); g.owedyk@gumed.edu.pl (G.O.); 6 Department of Emergency Medicine, Medical University of Gdańsk, University Clinical Centre in Gdańsk, 80-952 Gdańsk, Poland

**Keywords:** chronic limb-threatening ischemia, sarcopenia, psoas muscle area, psoas muscle density, psoas muscle index, computed tomography, revascularization, outcomes

## Abstract

**Background:** Sarcopenia is linked with high rates of adverse surgical outcomes, and computed tomography angiography (CTA)-based psoas measurements are used as imaging sarcopenia surrogates. Their prognostic value in patients with chronic limb-threatening ischemia (CLTI) undergoing revascularization remains uncertain. **Objectives:** To evaluate whether CTA-derived psoas muscle indices predict complications and mortality after lower-limb revascularization for CLTI. **Methods:** We performed a retrospective cohort study of consecutive adults who underwent open, hybrid, or endovascular revascularization for CLTI at a single tertiary center (March 2018–December 2021). Psoas muscle area (PMA) and density (PMD) were measured preoperatively on CTA at the mid-L3 vertebral level. Psoas muscle index (PMI) was calculated as PMA/height^2^. Patients were stratified by tertiles for each index (lowest tertile = “sarcopenic” vs. upper two tertiles). Outcomes included early in-hospital complications, late complications, overall complications, late mortality, and overall mortality. Group comparisons used χ^2^/Fisher tests with false discovery rate (FDR) adjustment; multivariable logistic regression with AIC-guided selection assessed independent predictors. **Results:** A total of 234 patients were included (median age 68 years; 65.4% men). Early complications occurred in 15.8%; late complications in 70.3%; overall mortality during follow-up was 26.6% (38/143 within follow-up data). In tertile analyses, none of the psoas-derived measures were significantly associated with early complications, late complications, overall complications, or mortality after FDR correction. Lower PMD showed consistent but non-significant trends toward higher late complications (84% vs. 64%), overall complications (87% vs. 72%), overall mortality (38% vs. 21%), and late mortality (37% vs. 20%) (all *p* < 0.05 unadjusted; all p_adj ≥ 0.139). In multivariable models, PMA, PMD, and PMI were not independent predictors of any outcome. **Conclusions**: In this retrospective cohort study, preoperative CTA-derived psoas indices were not independent predictors of early, late, or overall complications, nor of in-hospital or follow-up mortality after revascularization for chronic limb-threatening ischemia. Although lower psoas muscle density showed consistent trends toward higher risk, these associations did not reach statistical significance after adjustment. Taken together, our findings suggest that psoas-based measures have limited prognostic value in this setting and should be interpreted cautiously, while their potential role warrants confirmation in larger, prospective studies.

## 1. Introduction 

Chronic limb-threatening ischemia (CLTI) is the most advanced stage of peripheral arterial disease. It is characterized by insufficient blood supply to the limbs, leading to rest pain, non-healing ulcers and functional impairment [1]. In patients with CLTI, vascular procedures are often performed to improve blood flow, alleviate symptoms and save the limb [2]. These procedures are associated with various complications, both early and late, which can affect treatment outcomes and patients’ quality of life [3]. Complications such as wound healing impairment, pneumonia, acute kidney injury, infections, myocardial infarction, or stroke are frequent and significantly impact recovery and survival after vascular surgery.

Sarcopenia, characterized by reduced muscle mass, strength, and function [4], is an important prognostic factor for poor treatment outcomes or the occurrence of perioperative complications in surgical patients [5,6,7]. Several CT-derived markers have been proposed to indicate sarcopenia. It is important to note, however, that according to the EWGSOP2 consensus, imaging alone is insufficient for a clinical diagnosis. Sarcopenia requires not only reduced muscle mass, but also impaired muscle strength and/or function. Therefore, CT-based indices should be regarded as surrogate markers of muscle status rather than definitive diagnostic criteria. For the purposes of this study, we refer to these imaging measures as ‘sarcopenia markers’. These include skeletal muscle area and skeletal muscle index (SMA/SMI), subcutaneous fat index (SFI), and psoas-based parameters. In vascular surgery, psoas-based measures: Psoas Muscle Area (PMA), Psoas Muscle Density (PMD), and Psoas Muscle Index (PMI) are among the most frequently applied CT-derived muscle measurements in vascular surgery. They can be extracted directly from routine preoperative CTA without the need for additional software or labor-intensive segmentation, which makes them practical and reproducible. Focusing on these indices facilitates comparison with the existing vascular surgery literature [8].

Sarcopenia occurs in 30 to 45% of patients with peripheral arterial disease and chronic limb threatening ischemia [9,10,11,12]. Factors contributing to the development of sarcopenia in this group of patients include chronic inflammation, oxidative stress and limited physical activity [13]. Studies involving patients undergoing endovascular repair of abdominal aortic aneurysms and patients with colorectal cancer and pancreatic cancer provide evidence that the psoas muscle correlates with the incidence of complications and long-term survival [14,15,16,17,18]. The usefulness of psoas muscle-related indicators in the CLTI patient group requires further study due to the lack of consistent results in studies conducted to date [11,19].

Considering the usefulness of psoas muscle indices in assessing perioperative complications and long-term survival in other surgical populations, we hypothesized that lower preoperative values of these indices would be a predictor of mortality and morbidity after revascularization in the group of patients with CLTI.

The main objective of our study was to examine the relationship between psoas muscle indices and overall mortality in patients with CLTI undergoing vascular surgery. The second objective was to assess the relationship between these markers and the occurrence of postoperative complications, including myocardial infarction, stroke, revision surgery, limb amputation, and surgical site infection.

## 2. Methods

### 2.1. Study Design and Population

A retrospective analysis was conducted, including all consecutive patients who underwent lower limb revascularization for CLTI at the Vascular Surgery Department, University Clinical Center, Gdansk, Poland, between 1 March 2018 and 31 December 2021.

Inclusion criteria were adult patients (≥18 years old) who underwent primary revascularization for CLTI (Rutherford categories 4–5). Available preoperative computed tomography angiography (CTA) images, including imaging at the L3 vertebral level. 

Exclusion criteria included surgery for acute emergencies (e.g., acute limb ischemia), absence of preoperative CTA in our center’s records, incomplete clinical data, or prior major amputation of the index limb before revascularization. Patients undergoing revascularization in an elective (scheduled admission from outpatient clinic) or urgent setting (admission via emergency department or ward with surgery performed within days, but not immediate/emergency) were eligible.

This study was conducted in accordance with the Declaration of Helsinki and approved by the Institutional Ethics Committee (Independent Bioethical Committee of the Medical University of Gdansk, protocol code KB/340/2024; date of approval: 5 July 2024).

### 2.2. Data Collection and Study Variables

Based on patients’ medical records, we collected data on demographic characteristics, comorbidities, operative urgency (elective vs. urgent), and procedural details (open, hybrid, or endovascular). Postoperative outcomes were extracted from the institutional vascular registry and electronic medical records, including follow-up visits in the outpatient clinic.

Psoas muscle measurements: PMA, PMD were obtained as described below. The PMI was subsequently calculated from PMA.

### 2.3. Assessment of Sarcopenia Markers

**Figure 1:**
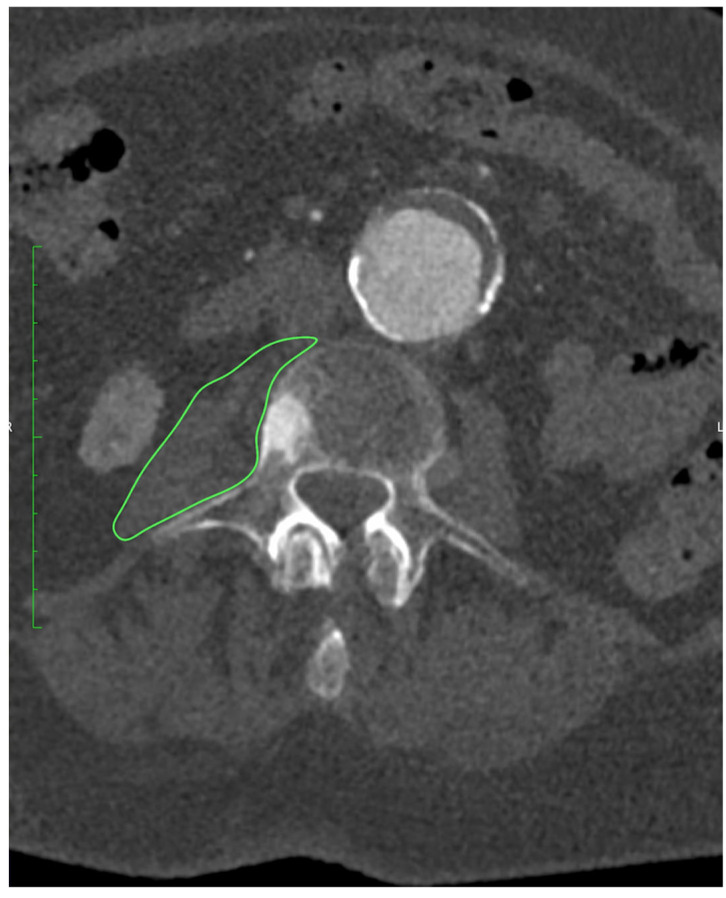
Measurement of PMA at the level of L3 using OsiriX MD (Pixmeo SARL, Geneva, Switzerland), version 14.1.0 software.

All patients underwent preoperative CTA of the aorto-iliac and femoral arteries performed at our institution. Examinations were acquired on a 128-slice scanner (SOMATOM X.cceed, Siemens Healthineers, Erlangen, Germany) using a standardized contrast-enhanced protocol (100–120 mL of iodinated contrast, injection rate 4–5 mL/s, bolus tracking in the abdominal aorta). Images were reconstructed with a slice thickness of 0.5 or 1.0 mm, depending on the reconstruction protocol used. All scans were obtained on the same scanner model. All measurements were performed preoperatively on CTA at the mid-L3 vertebral level using standardized axial sections in the arterial phase. On a representative slice, both psoas muscles were manually delineated with dedicated imaging software OsiriX MD (Pixmeo SARL, Geneva, Switzerland), version 14.1.0, and their cross-sectional surface areas (PMA, cm^2^) were recorded, as presented on Figure 1. From the same regions of interest, the average PMD (Hounsfield units) was determined. The PMI (cm^2^/m^2^) was derived by normalizing the combined PMA to the patient’s height squared.

### 2.4. Statistical Analysis

Continuous variables were expressed as mean and median with range. Categorical variables were presented as counts and percentages. To account for multiple comparisons, raw *p*-values from bivariate analyses (Table 4) were adjusted using the Benjamini–Hochberg false discovery rate (FDR) procedure, yielding the reported p_adjusted values. The dataset was stratified by tertiles of PMA, PMD, and PMI in such a way that the one-third of patients with the lowest PMA/PMD/PMI values was classified as the ‘sarcopenic’ group, while the remaining two-thirds were treated as the control group. Following this stratification, a series of comparisons between the sarcopenic and control groups was conducted to identify differences in clinical outcomes. Depending on the met assumptions, either the χ^2^ test or Fisher’s exact test was applied. 

The primary objective of the regression modeling was to explore the predictive value of imaging-derived muscle parameters for five different outcomes: early complications, late complications, overall complications, late mortality and overall mortality. To minimize the impact of highly influential observations, we first fitted an initial logistic regression models to the full dataset and calculated Cook’s distances, applying a 4/*n* cutoff. Observations exceeding this threshold were excluded, and the filtered dataset was used for all subsequent analyses. 

Multicollinearity among predictors was evaluated using the variance inflation factor (VIF), and predictors with VIF values greater than 10 were excluded from the models. Candidate predictors included: procedure type, age, sex, comorbidities (hypertension, diabetes, chronic kidney disease, dialysis therapy, history of myocardial infarction, neurological disease), smoking status, previous vascular interventions, and one imaging-derived muscle parameter (PMA, PMI, PMD). Bidirectional stepwise selection based on the Akaike Information Criterion (AIC) was applied to identify the optimal subset of predictors for each key parameter. The predictors retained after this selection process were used in the final regression models. Results from the logistic regression models were reported as β coefficients with standard errors, *z* values, *p* values, odds ratio, and relative change in odds. 

In addition to overall analyses, subgroup analyses were performed according to procedure type (open, hybrid, endovascular) and sex.

All analyses were conducted in R (version 4.1) using the stats [20] package for regression fitting, Cook’s distance, χ^2^, Fisher’s exact test; MASS [21] for AIC-based stepwise selection; car [22] for multicollinearity assessment; and ggplot2 [23] for all graphical outputs. 

## 3. Results

### 3.1. Baseline Characteristics

A total of 234 patients were analyzed. Median age was 68 years (range 52–93), and 65.4% were men. Median BMI was 25 kg/m^2^ (range 14–53). Hypertension was present in 64.5% of patients, diabetes mellitus in 43.2%, and coronary artery disease in 26.1%. Other comorbidities are summarized in Table 1.

### 3.2. Psoas Muscle Measurements

Median PMA was 11.0 cm^2^ (range 4.9–21.1), median PMD was 37.5 HU (range 8.8–75.2), and median PMI was 3.8 cm^2^/m^2^ (range 1.6–6.9). Detailed distributions are provided in Table 2. The cutoff values defining the lowest tertiles were PMA ≤ 8.87 cm^2^, PMI ≤ 3.27 cm^2^/m^2^, and PMD ≤ 31.97 HU.

### 3.3. Clinical Outcomes

Early postoperative complications occurred in 37 patients (15.8%), with reoperation required in 21 (9.0%), wound infection in 5 (2.1%), and in-hospital death in 4 (1.7%). Other early adverse events are detailed in Table 3.

During follow-up, late complications were observed in 116 patients (70.3%), including reintervention in 77 (47.0%), major amputation in 17 (12.2%), myocardial infarction in 8 (5.8%), and stroke in 8 (5.8%). Overall mortality during follow-up was 26.6% (38/143). Detailed late outcomes are summarized in Table 3.

### 3.4. Tertile-Based Analysis

When stratified by tertiles of PMA, PMD, and PMI, no statistically significant associations were found between these markers and the occurrence of early complications, late complications, overall complications, or mortality after adjustment for multiple comparisons.

For PMD, patients in the lowest tertile had consistently higher adverse event rates compared with those in higher tertiles: late complications occurred in 84% vs. 64% (*p* = 0.011; p_adj = 0.139), overall complications in 87% vs. 72% (*p* = 0.025; p_adj = 0.139), overall mortality in 38% vs. 21% (*p* = 0.026; p_adj = 0.139), and late mortality in 37% vs. 20% (*p* = 0.028; p_adj = 0.139).

For PMA, no meaningful associations were observed (all *p* > 0.08). PMI showed a borderline relationship with late mortality (34% vs. 19%, *p* = 0.051; p_adj = 0.205), but no other outcomes were significantly affected. Detailed results of the tertile analysis are presented in Table 4. Stratified analyses by sex and procedure type are presented in Supplementary Table S1. Although some numerical differences were observed, for example, higher rates of late complications and mortality in male patients with lower PMD, none of these associations reached statistical significance after correction for multiple testing.

### 3.5. Regression Analyses

In multivariate logistic regression, none of the psoas-derived parameters (PMA, PMD, or PMI) were independently associated with early complications, late complications, or overall adverse outcomes (all *p* > 0.1). Similarly, no significant relationship was observed between psoas muscle markers and overall or late mortality.

By contrast, conventional clinical predictors demonstrated stronger prognostic value. The presence of chronic heart failure was independently associated with more than a 15-fold increase in overall mortality risk (OR 15.5, 95% CI 3.2–74.8; *p* < 0.001), while age showed a borderline association, with each additional year increasing mortality risk by approximately 5% (OR 1.05, 95% CI 1.00–1.10; *p* = 0.054). In our tertile-based analyses (Table 4), patients with lower PMD and PMI showed consistently higher absolute rates of late complications and mortality (e.g., PMD: 84% vs. 64% late complications; 38% vs. 21% overall mortality), even though these associations lost statistical significance after FDR adjustment. This pattern indicates a potential prognostic signal, but its effect was attenuated in adjusted regression models. Detailed regression outputs for psoas muscle density are presented in Supplementary Tables S2 and S3.

While PMD itself was not an independent predictor of complications or mortality, previous vascular interventions and smoking were associated with higher complication risk, and heart failure and age remained the strongest determinants of mortality.

## 4. Discussion

CLTI represents the most advanced stage of PAD and is strongly linked to high rates of mortality, major amputation, and impaired quality of life [1,24]. With an aging population, persistently high smoking rates (in Poland), and the growing burden of comorbidities such as diabetes, hypercholesterolemia, chronic kidney disease, coronary artery disease, and heart failure, the number of patients living with CLTI is expected to rise [25,26]. What makes CLTI particularly concerning is its prognosis: one-year mortality reaches 20–40%, and major amputation is as frequent [27]. Late complications such as reintervention, myocardial infarction, or stroke can affect as many as 70–80% of patients. Despite advances in specialized vascular care, the risk of serious adverse outcomes remains high [28]. Complications may appear early as perioperative events, unplanned readmissions, or later, as limb loss and progressive decline in quality of life. Beyond its clinical consequences, CLTI also carries a heavy socioeconomic burden, driving up healthcare costs and resource use [29]. Taken together, these factors underline the urgent need for reliable prognostic markers to help stratify risk and guide management strategies.

The use of parameters for assessing the psoas muscle in CT has many advantages: they do not require additional financial outlay, the measurements are available as a standard preoperative imaging protocol, there is no need for additional tests, the results are objective and can be useful in predicting postoperative outcomes [30,31]. 

In other surgical fields, psoas-based measurements have been associated with adverse outcomes, for example, higher mortality after AAA repair, increased morbidity after major oncological resections, or worse survival after TAVI. These data support the concept that muscle parameters may reflect overall physiological reserve and vulnerability to surgical stress [15,32]. Psoas muscle measurements may reflect a physiological reserve that is not considered in conventional risk scales, especially among older patients. In patients undergoing major cancer resections, a reduction in psoas muscle area or density predicts an increased risk of perioperative infections, increased incidence of thromboembolic events, longer hospital stays, and overall morbidity. In addition, patients undergoing liver and colon resection with radiologically confirmed sarcopenia are at a significantly higher risk of complications and require more intensive postoperative monitoring [33,34].

Cardiac surgery is another field in which psoas muscle measurements demonstrate clinical utility. Patients with reduced psoas muscle area undergoing both open and transcatheter aortic valve implantation (TAVI) required prolonged mechanical ventilation, had serious morbidity, longer hospital stays, and higher costs were observed [35]. Among patients undergoing TAVI, sarcopenia, defined as a low PMA index, was independently associated with higher overall and cardiac mortality [36,37]. These findings are consistent with the concept that muscle parameters reflect overall physiological resilience and the ability to withstand the high stress associated with surgery.

The potential clinical applications of reliable markers of sarcopenia in CLTI remain theoretically compelling. Accurate preoperative risk stratification could enable the implementation of targeted prehabilitation programs involving structured exercise therapy and nutritional supplementation [38]. Furthermore, objective muscle assessment could aid in the choice of treatment between aggressive revascularization and conservative treatment in patients with borderline indications for surgery. Despite evidence from other surgical fields, our study did not find a significant correlation between psoas muscle markers and clinical outcomes in patients with chronic limb-threatening ischemia who underwent revascularization. In the tertile-based analysis, no psoas-derived parameter demonstrated a statistically significant association with early complications, late complications, overall complications, or mortality after correction for multiple comparisons. Nevertheless, several trends emerged that may be clinically relevant. For PMD, patients in the lowest tertile had markedly higher event rates: late complications occurred in 84% versus 64% in the higher tertiles (χ^2^ = 6.45, *p* = 0.011; p_adj = 0.139), overall complications in 87% versus 72% (*p* = 0.025; p_adj = 0.139), overall mortality in 38% versus 21% (*p* = 0.026; p_adj = 0.139), and late mortality in 37% versus 20% (*p* = 0.028; p_adj = 0.139). Although these associations lost statistical significance after adjustment, the consistent direction of effect suggests that PMD may be a more reliable indicator of muscle quality than size-based indices. In contrast, PMA did not show any meaningful association with outcomes (all *p* > 0.08). This limitation is not surprising, as absolute muscle area is strongly confounded by body size and sex: a small, physically fit woman will naturally have a smaller muscle area than an obese, chronically ill man, yet the prognostic implications are the opposite. PMI, which normalizes PMA to body height, was expected to mitigate this issue. However, in our cohort, it did not reach significance; the closest finding was for late mortality (34% vs. 19% between the lowest and higher tertiles, *p* = 0.051; p_adj = 0.205). This may reflect limited statistical power, and larger sample sizes will be necessary to clarify whether PMI holds prognostic value. Subgroup analyses stratified by sex and procedure type did not reveal any statistically significant associations between psoas-derived markers and outcomes after correction for multiple testing (Supplementary Table S1).

In multivariate logistic models, none of the psoas-derived parameters (PMA, PMD, or PMI) emerged as independent predictors of early or late complications, nor of overall or late mortality. Although lower PMD and PMI consistently trended toward higher risk, these effects were attenuated after adjustment for covariates. By contrast, conventional clinical factors retained strong prognostic significance: the presence of chronic heart failure increased overall mortality more than fifteen-fold (OR 15.5, 95% CI 3.2–74.8; *p* < 0.001), and age showed a borderline association, with a 5% increase in mortality risk per additional year (OR 1.05, 95% CI 1.00–1.10; *p* = 0.054). In regression models restricted to PMD, several associations emerged. Patients with a history of previous vascular interventions had over a threefold higher risk of late complications (OR 3.16, 95% CI 1.4–7.1; *p* = 0.007), and those with chronic heart failure showed a strong but borderline-significant increase in complication risk (OR 6.76, 95% CI 0.8–54.4; *p* = 0.089). For mortality, PMD itself was not an independent predictor, but the same models highlighted established risk factors: heart failure conferred more than a fifteen-fold increase in mortality risk (OR 15.5, 95% CI 3.2–74.8; *p* < 0.001), and age demonstrated a borderline effect, with each additional year raising the risk of death by ~5% (*p* = 0.03–0.05). Overall, our results suggest that psoas-based indices, although attractive due to their simplicity and availability in routine CT imaging, do not show consistent prognostic value in the CLTI population. Although PMD showed some trends towards a higher risk of late complications and mortality, these associations lost significance after adjustment and were clearly overshadowed by established clinical determinants such as age and comorbidity, particularly heart failure. This emphasizes an important limitation of looking at muscle measurements in isolation: at the moment they do not appear to be robust enough to serve as independent predictors of early, late, or overall complications, or of mortality (both in-hospital and during follow-up) in patients with CLTI. Instead, their role may be more supportive, for example, as part of a multidimensional assessment of frailty or nutritional status, rather than as a prognostic tool.

## 5. Limitations

This study has several limitations. First, mortality could only be analyzed as a binary outcome (“death during follow-up”) since exact dates and causes of death were unavailable for patients dying outside our institution, precluding formal survival analysis. Second, we applied false discovery rate (FDR) correction to account for multiple comparisons, which may have obscured potentially relevant associations in this moderate-sized cohort, particularly for PMD. Third, although whole-skeletal muscle indices (e.g., SMA, SMI) are regarded as more representative of global sarcopenia, we focused on psoas-based markers because they are widely used in vascular surgery research and easily derived from routine preoperative CTA; nevertheless, future studies should also evaluate SMA/SMI and fat-related indices. Fourth, we assessed only preoperative values and did not explore postoperative changes, which may also carry prognostic significance. Fifth, PMD was measured on arterial-phase contrast-enhanced CTA, which systematically elevates muscle density values compared with non-contrast imaging and limits direct comparability with prior studies. Finally, sarcopenia was defined using tertile-based cutoffs, an approach commonly used in vascular research but is still arbitrary; standardized thresholds are needed to improve comparability across studies.

## 6. Conclusions

In this retrospective exploratory analysis, psoas muscle indices were not identified as independent predictors of complications or mortality after revascularization for chronic limb-threatening ischemia. While psoas-derived measures did not retain statistical significance after adjustment for confounders and multiple comparisons, consistent trends were observed for lower psoas muscle density, suggesting a potential prognostic signal. These findings should therefore be interpreted cautiously. Our results highlight the limited and uncertain role of psoas metrics in this population. Future large-scale, prospective studies with comprehensive survival data are needed to determine whether these imaging-based measures add value when integrated into multidimensional frailty and nutritional assessments.

## Figures and Tables

**Table 1:** Baseline characteristics of the study population. Values are presented as median (range) for continuous variables and number (percentage) for categorical variables.

Characteristic	Value	%
Age, years	Median 68 (range 52–93)	
Male sex	153	65.4
BMI, kg/m^2^	Median 25 (range 14–53)	
Hypertension	151	64.5
Diabetes mellitus	101	43.2
Coronary artery disease	61	26.1
Previous myocardial infarction	38	16.2
Previous vascular intervention	85	36.5
Chronic heart failure	19	8.1
Chronic kidney disease (eGFR < 30)	19	8.2
Dialysis therapy	7	3.0
Neurological comorbidity	29	12.4
History of smoking	166	70.9
Length of hospital stay, days	Median 6 (range 1–82)	

**Table 2:** Psoas muscle measurements. Values are presented as median (range). PMA—psoas muscle area; PMD—psoas muscle density; PMI—psoas muscle index normalized to height squared.

Variable	Value
PMA, cm^2^	11.0 (4.9–21.1)
PMD, HU	37.5 (8.8–75.2)
PMI, cm^2^/m^2^	3.8 (1.6–6.9)

**Table 3:** Clinical outcomes. Early complications included reoperation, wound infection, and in-hospital death, with additional events categorized as “other.” Late complications comprised reintervention, major amputation, myocardial infarction, and stroke. Mortality was reported separately as in-hospital death and overall mortality during follow-up.

Outcome	Value	%
Early complications (any)	37	15.8
Reoperation (early)	21	9.0
Wound infection (early)	5	2.1
In-hospital death	4	1.7
Other early complications	14	6.0
Late complications (any)	116	70.3
Reintervention (late)	77	47.0
Major amputation (late)	17	12.2
Myocardial infarction (late)	8	5.8
Stroke (late)	8	5.8
Overall mortality (follow-up)	38	26.6

**Table 4:** Tertile-based analysis of psoas measurements and clinical outcomes. Values are presented as percentage of events in the lowest versus higher tertiles. Comparisons were performed using the chi-square test. p_adj refers to *p*-value corrected for multiple comparisons. PMA—psoas muscle area; PMD—psoas muscle density; PMI—psoas muscle index.

Marker	Outcome	Comparison (Low vs. High)	Test/χ^2^	*p*	p_adj
PMA	Early complications	13% vs. 18%	χ^2^ = 1.61	0.205	0.410
PMA	Late complications	59% vs. 71%	χ^2^ = 0.78	0.378	0.473
PMA	Late mortality	29% vs. 21%	χ^2^ = 0.84	0.359	0.473
PMA	Overall complications	69% vs. 81%	χ^2^ = 2.89	0.089	0.279
PMA	Overall mortality	30% vs. 25%	χ^2^ = 0.42	0.519	0.611
PMD	Early complications	13% vs. 17%	χ^2^ = 0.79	0.375	0.473
PMD	Late complications	84% vs. 64%	χ^2^ = 6.45	0.011	0.139
PMD	Late mortality	37% vs. 20%	χ^2^ = 4.84	0.028	0.139
PMD	Overall complications	87% vs. 72%	χ^2^ = 5.00	0.025	0.139
PMD	Overall mortality	38% vs. 21%	χ^2^ = 4.93	0.026	0.139
PMI	Early complications	12% vs. 18%	χ^2^ = 1.42	0.234	0.425
PMI	Late complications	57% vs. 69%	χ^2^ = 0.11	0.743	0.743
PMI	Late mortality	34% vs. 19%	χ^2^ = 3.80	0.051	0.205
PMI	Overall complications	78% vs. 78%	χ^2^ = 0.21	0.645	0.716
PMI	Overall mortality	34% vs. 23%	χ^2^ = 2.74	0.098	0.279

## Data Availability

Data supporting the findings of this study are available from the corresponding author upon reasonable request.

## Supplementary Materials

The following supporting information can be downloaded at: https://www.mdpi.com/article/10.3390/medsci13040227/s1, Table S1: Stratified tertile analysis of psoas measurements (PMA, PMD, PMI) by sex and procedure type. Table S2: Multivariate logistic regression model for psoas muscle density (PMD) and overall complications. Table S3: Multivariate logistic regression model for PMD and overall mortality.
