# Supplementary material for: Limited Prognostic Value of Psoas Muscle Indices in Patients Undergoing Revascularization for Chronic Limb-Threatening Ischemia
Source: Med Sci (Basel). 2025 Oct 12;13(4):227. doi: 10.3390/medsci13040227 (PMC12550900; doi:10.3390/medsci13040227)
Supplement: Supplementary file 1 [file medsci-13-00227-s001.zip › medsci-3889708-supplementary.pdf]

Table S1. Stratified tertile analysis of psoas measurements (PMA, PMD, PMI) by sex and procedure type. Values are presented as percentage of events in the lowest versus higher tertiles, with corresponding adjusted p-values (p_adj). Subgroups include female and male patients, as well as open, hybrid, and endovascular procedures. No statistically significant associations were observed after correction for multiple comparisons.

Marker	Subgroup	Complications_early (Low vs High %)	Complications_late (Low vs High %)	Complications_overall (Low vs High %)	Death_late (Low vs High %)	Death_overall (Low vs High %)	Complications_early (p_adj)	Complications_late (p_adj)	Complications_overall (p_adj)	Death_late (p_adj)	Death_overall (p_adj)
PMA	Female patients	11.1% vs 22.2%	59.6% vs 76.9%	63.3% vs 86.7%	30.2% vs 30.8%	30.2% vs 30.8%	0.913	0.913	0.779	1.000	1.000
PMA	Male patients	13.3% vs 17.4%	91.7% vs 72.0%	92.3% vs 79.8%	27.3% vs 21.3%	27.3% vs 23.7%	1.000	0.451	0.651	0.803	0.803
PMA	Open procedures	9.8% vs 16.3%	67.7% vs 74.5%	72.7% vs 81.0%	29.6% vs 27.7%	29.6% vs 29.2%	0.596	0.666	0.596	0.901	0.966
PMA	Hybrid procedures	9.1% vs 23.8%	75.0% vs 64.3%	75.0% vs 70.6%	37.5% vs 27.3%	37.5% vs 27.3%	1.000	1.000	1.000	1.000	1.000
PMA	Endovascular procedures	15.4% vs 18.4%	60.0% vs 73.0%	61.9% vs 84.6%	26.3% vs 13.3%	26.3% vs 16.7%	1.000	0.787	0.606	0.787	0.800
PMD	Female patients	9.7% vs 16.0%	85.0% vs 52.5%	86.4% vs 59.5%	36.8% vs 27.0%	36.8% vs 27.0%	0.913	0.276	0.278	0.913	0.913
PMD	Male patients	14.9% vs 17.9%	83.3% vs 70.7%	87.9% vs 78.5%	37.0% vs 15.3%	39.3% vs 16.9%	0.803	0.451	0.491	0.159	0.159
PMD	Open procedures	15.2% vs 13.6%	81.5% vs 67.8%	87.1% vs 73.3%	41.7% vs 22.0%	44.0% vs 22.0%	0.888	0.596	0.596	0.596	0.596
PMD	Hybrid procedures	22.2% vs 17.4%	100.0% vs 58.8%	100.0% vs 63.2%	40.0% vs 28.6%	40.0% vs 28.6%	1.000	1.000	1.000	1.000	1.000
PMD	Endovascular procedures	4.3% vs 23.1%	83.3% vs 61.5%	83.3% vs 73.8%	29.4% vs 12.5%	29.4% vs 15.6%	0.606	0.666	0.800	0.787	0.787

PMI	Female patients	12.0% vs 13.8%	63.2% vs 61.9%	67.5% vs 69.6%	32.4% vs 23.8%	32.4% vs 23.8%	1.000	1.000	1.000	0.913	0.913
PMI	Male patients	10.7% vs 18.4%	86.4% vs 71.1%	87.0% vs 79.8%	36.8% vs 17.9%	36.8% vs 20.6%	0.651	0.451	0.743	0.451	0.491
PMI	Open procedures	9.8% vs 16.3%	68.8% vs 74.1%	73.5% vs 80.7%	35.7% vs 23.9%	35.7% vs 25.5%	0.596	0.700	0.641	0.596	0.596
PMI	Hybrid procedures	15.4% vs 22.2%	80.0% vs 58.3%	80.0% vs 66.7%	44.4% vs 20.0%	44.4% vs 20.0%	1.000	1.000	1.000	1.000	1.000
PMI	Endovascular procedures	12.5% vs 18.0%	72.2% vs 65.8%	73.7% vs 77.5%	25.0% vs 12.5%	25.0% vs 15.6%	0.837	0.837	0.837	0.800	0.800

Table S2. Multivariate logistic regression model for psoas muscle density (PMD) and overall complications. Values are expressed as regression coefficients (Estimate), standard error (Std. Error), z-value, p-value, odds ratio (OR), and relative change in odds (%). None of the psoas-derived parameters reached statistical significance. Previous vascular interventions (OR 3.16, p=0.007) and smoking (OR 2.51, p=0.041) were independently associated with increased risk of complications, while heart failure showed a borderline effect (OR 6.76, p=0.089).

Term	Estimate	Std_Error	z_value	p_value	OR	Δ odds %
**(Intercept)**	**-0.77**	**0.49**	**-1.57**	**0.12**	**0.46**	**-53.71**
**Previous_interventions**	**1.15**	**0.42**	**2.71**	**0.01**	**3.16**	**216.29**
**Heart failure**	**1.91**	**1.12**	**1.7**	**0.09**	**6.76**	**575.85**
**Male_sex**	**0.58**	**0.39**	**1.49**	**0.14**	**1.78**	**78.47**
**Smoking**	**0.92**	**0.45**	**2.05**	**0.04**	**2.51**	**151.13**
**Neurological comorbidity**	**0.96**	**0.7**	**1.37**	**0.17**	**2.61**	**160.9**

Table S3. Multivariate logistic regression model for PMD and overall mortality. Values are expressed as regression coefficients (Estimate), standard error (Std. Error), z-value, p-value, odds ratio (OR), and relative change in odds (%). PMD itself was not an independent predictor. Among clinical covariates, chronic heart failure was strongly associated with mortality (OR 15.49, p<0.001), while age showed a borderline association (OR 1.05 per year, p=0.054).

Term	Estimate	Std_Error	z_value	p_value	OR	Δ odds %
**(Intercept)**	**-4.65**	**1.76**	**-2.64**	**0.01**	**0.01**	**-99.05**
**Heart failure**	**2.74**	**0.82**	**3.33**	**0.0**	**15.49**	**1448.71**
**Age**	**0.05**	**0.02**	**1.93**	**0.05**	**1.05**	**4.9**
